# Vis-NIR soil spectral library of the Hungarian Soil Degradation Observation System

**DOI:** 10.1038/s41597-025-04667-9

**Published:** 2025-03-01

**Authors:** János Mészáros, Zsófia Kovács, Péter László, Szilvia Vass-Meyndt, Sándor Koós, Béla Pirkó, Nóra Szűcs-Vásárhelyi, Zsófia Bakacsi, Annamária Laborczi, Kitti Balog, László Pásztor

**Affiliations:** 1 HUN-REN Centre for Agricultural Research, Institute for Soil Sciences, Department of Soil Mapping and Environmental Informatics, Budapest, Hungary; 2 HUN-REN Centre for Agricultural Research, Institute for Soil Sciences, Department of Soil Chemistry and Material Turnover, Budapest, Hungary; 3 HUN-REN Centre for Agricultural Research, Institute for Soil Sciences, Department of Soil Physics and Water Management, Budapest, Hungary

**Keywords:** Agriculture, Geography, Environmental monitoring, Environmental impact

## Abstract

Since soil spectroscopy is considered to be a fast, simple, accurate and non-destructive analytical method, its application can be integrated with wet analysis as an alternative. Therefore, development of national-level soil spectral libraries containing information about all soil types represented in a country is continuously increasing to serve as a basis for calibrated predictive models capable of assessing physical and chemical parameters of soils at multiple spatial scales. In this article, we present a database containing laboratory and visible-near infrared spectral data of legacy soil samples from the Hungarian Soil Degradation Observation System (HSDOS). The published data set includes the following parameters measured in 5,490 soil samples: pH_KCl_, soil organic matter (SOM), calcium carbonate (CaCO_3_), total salt content (TSC), total nitrogen (TN), soluble phosphorus (P_2_O_5_-AL), soluble potassium (K_2_O-AL), plasticity index according to Hungarian standard (PLI), soil profile depth and reflectance data between 350 and 2,500 nm wavelength. The presented database can be a complement for further soil related research on continental, national or regional scales to support sustainable soil management.

## Background & Summary

In the contemporary era, the pedosphere is regarded as a non-renewable resource^1^, which serves to reinforce the endeavours to mitigate the consequences of climate change^2^ and, through agricultural cultivation^3^, to enhance food security^4^. Consequently, the conservation of this resource represents one of the most significant environmental challenges, necessitating the monitoring of a multitude of physical and chemical parameters, usually measured in laboratories by cost and labour intensive techniques. Thus, in recent decades, cost-effective and non-destructive methods have been developed and increasingly used, including diffuse reflectance spectroscopy^5,6^ in the visible and near-infrared (Vis-NIR: 350–2,500 nm) or mid-infrared (MIR: 2,500–25,000 nm) regions of the electromagnetic spectrum^7^.

Therefore, using these techniques, soil spectral libraries (SSLs) containing soil spectra in one or both wavelength regions together with corresponding soil physical, chemical and biological properties have been developed for different geographical scales. Several global scale SSLs have been established, such as in the study of Brown *et al*.^8^ with soil samples from U.S. states, Africa, Asia, the Americas and Europe. Viscarra Rossel *et al*.^6^ also built up a global library representing 92 countries and seven continents. The Open Soil Spectral Library of Safanelli *et al*.^9^ is an example for openly available global SSL containing not only Vis-NIR and MIR spectral information and soil samples from U.S., Africa, Asia, the Americas, Antarctica and Europe but pre-compiled models also to test the inference of soil parameters.

On a national scale, several Vis-NIR libraries (both openly available or with limited access) have been implemented lately, i.e. in Brazil^10–12^ China^13^, Czech Republic^14^, Denmark^15^, France^16,17^, New Zealand^18^ and U.S.^19^. Very few studies have been published already about SSLs of soils in Hungary. Only MIR SSL^20^ has been developed recently, however it partially covers the country and is not openly available in data repositories.

To fulfill the hiatus of a Vis-NIR SSL for Hungarian soils, legacy samples of the Hungarian Soil Degradation Observation Sytem (HSDOS)^21^ have been measured spectrally in 2023 and joined with consecutive soil physical and chemical parameters.

HSDOS was established to collect, manage, analyse and publish soil data related to the state of soils together with the agriculture load (tillage, fertilisation, plant protection) by setting up an appropriate information system to support soil protection in Hungary as well as to inform publicity on the state and degradation processes of soils. The HSDOS provides information on the type and extent of soil degradation caused by different site pressures by distinguishing between representative farm types of agricultural practice in Hungary. Originally HSDOS was intended to operate as a monitoring system, but due to certain national and international constrains and step-backs it ended up in one campaign of soil sampling carried out in 2011. The collected soil samples have been stored in the Institute for Soil Sciences’ soil sample bank. To build the spectrum library and to evaluate spectral behaviour of Hungarian soils within the 350–2,500 nm range, 6,935 composite soil samples of 2,030 parcels from 294 representatively selected agricultural farms were measured.

The most promising benefit of the two united data sets is the significant extension of prediction possibilities to estimate important primary soil properties, e.g. soil organic carbon^22^, cation exchange capacity^23^, pH^24^, texture^25^ and base saturation^26^ in regional or national scale based on non-destructive spectral data collection. Potential users of this database are soil and environmental researchers who study and monitor the past and present condition of soils.

## Methods

In this section, the used methods during the field sampling, laboratory analysis and spectral measurements are presented in detail.

### Sampling strategy

Within the framework of HSDOS first representative farms were assigned. The 294 farms were selected from the database of the Hungarian Central Statistical Office, based on their relevance for the application of fertilizers, organic manure and pesticides, also considering (1) the types of loads, (2) geographical location (at county level) and soil type, and (3) the size of the farm. Composite soil sampling was carried out on the plots of the farms in 5 hectare representative sampling units (RSU) following strict protocol.

**Fig. 1:**
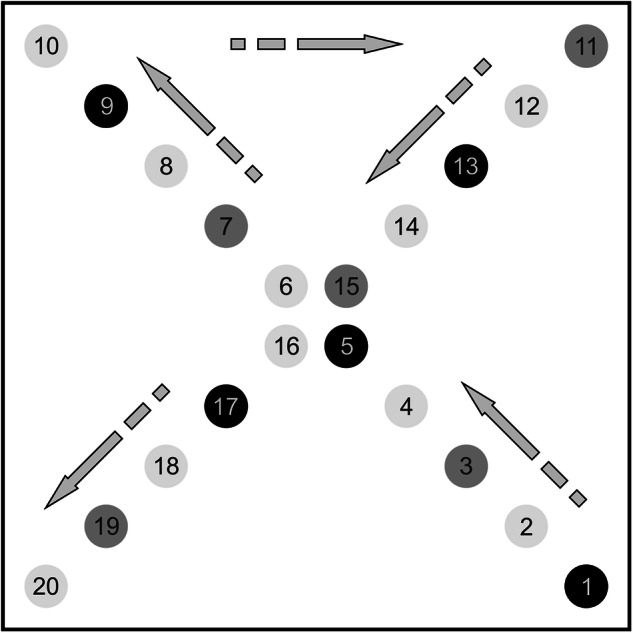
Sampling protocol for collecting composite soil samples in the 5-hectare representative sampling unit (RSU). The darker shade of grey is proportional to the number of sampled soil layers: one depth from 0–30 cm–light grey; two depths from 0–30 cm and 30–60 cm–middle grey; three depths from 0–30 cm, 30–60 cm and 60–90 cm–dark grey.

Collected soil data refers to soil cores drilled down to 90 cm, with 0–30, 30–60 and 60–90 cm equidistant sampling depths. Each 5-hectare area was surveyed using a cross-sampling method (Fig. 1), involving 20 samples of soil at depths of 0–30 cm, 10 samples at depths of 30–60 cm, and 5 samples at depths of 60–90 cm, forming composite samples from each soil depth. In detail, for the upper soil layer (0–30 cm), the average sample is formed from a total of 20 sample points by traversing 10–10 along the two diagonals of the designated RSU. The soil layer at a depth of 30–60 cm is sampled at every second sampling point location, resulting in a total of 10 sampling points along the two diagonals of the RSU, thus forming a composite sample. Every fourth sampling point, the soil layer at 60–90 cm depth is also sampled, resulting in a total of 5 sample points along the two diagonals of RSU for forming the composite sample.

**Fig. 2:**
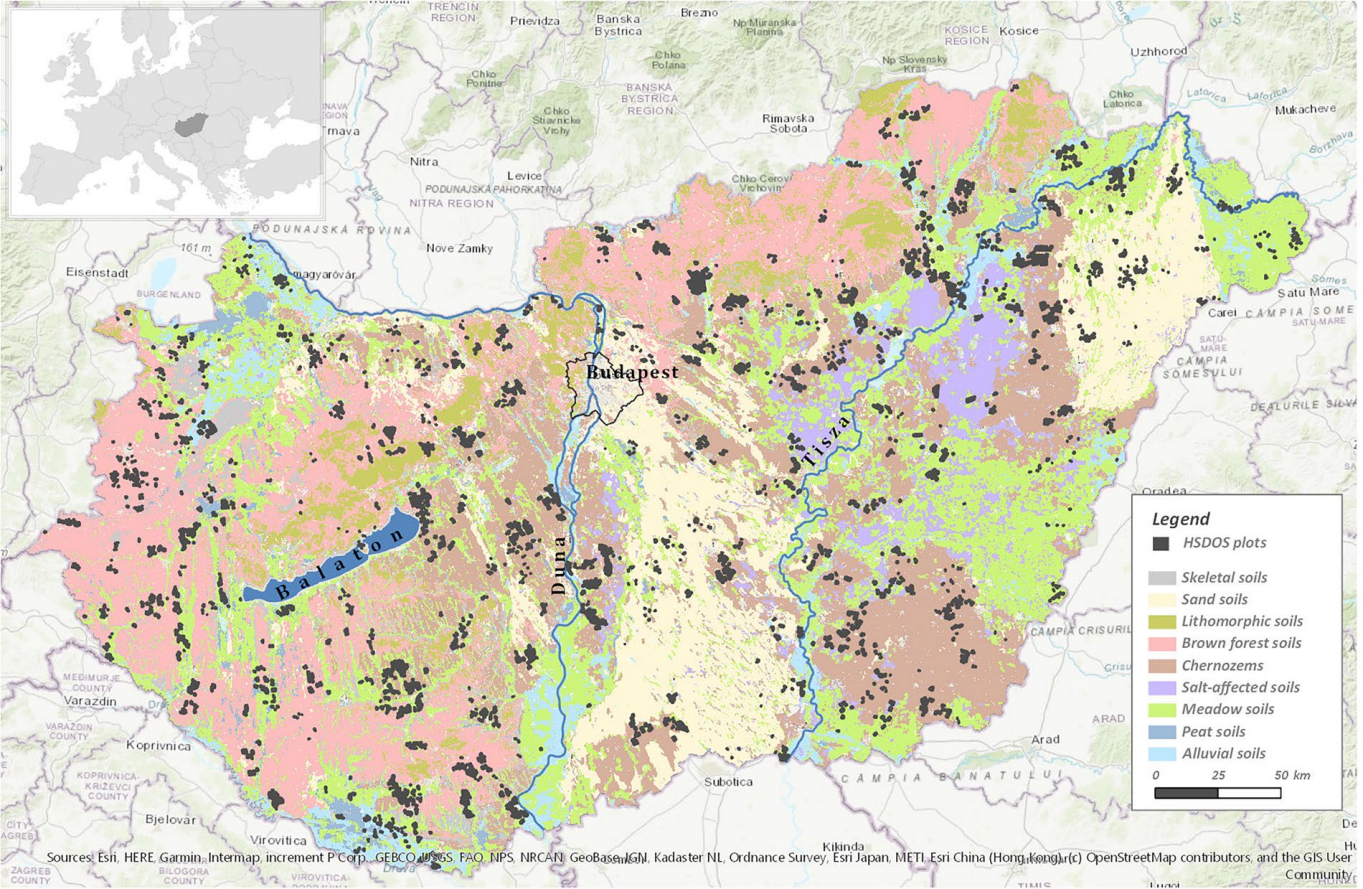
Spatial distribution of the HSDOS plots, where 5 hectare representative sampling units (RSU) were sampled, overlaid on the main soil type map of Hungary^47^.

RSUs are basically polygons. For georeferencing composite samples, the centroids of RSUs were generated in ArcGIS and defined by their Easting and Northing planar coordinates according to the Hungarian Unified National Projection System (EOV/HD72 – ID: 23700 in the EPSG Geodetic Parameter Data set^27^, see https://epsg.io/23700). The same planar coordinates were also transformed into the WGS84 ellipsoidal reference system (ID: 4326 in the EPSG Geodetic Parameter Data set^27^, see https://epsg.io/4326). Both types of coordinates were included in the data set. Due to the geographically stratified selection of representative farms, the spatial distribution of RSUs (Fig. 2) basically covers the full country, which together with the expert based selection of farms within counties guarantee a nationally representative soil observation system.

### Laboratory analysis

Composite soil samples were taken during the sampling campaigns in the spring (1. March-1. June) and summer-fall period (1. July-30. November) of 2011, and processed continuously after the sampling in accredited laboratory (Fejér County MGSZH Soil Testing Laboratory, Velence). The samples were measured for:

- soil chemical parameters: pH_KCl_, soil organic matter (SOM), calcium-carbonate (CaCO_3_) content, total salt content (TSC), total nitrogen (TN), soluble phosphorus (P_2_O_5_-AL), soluble potassium (K_2_O-AL)
- soil physical parameters: plasticity index according to Hungarian standard (PLI).

**Fig. 3:**
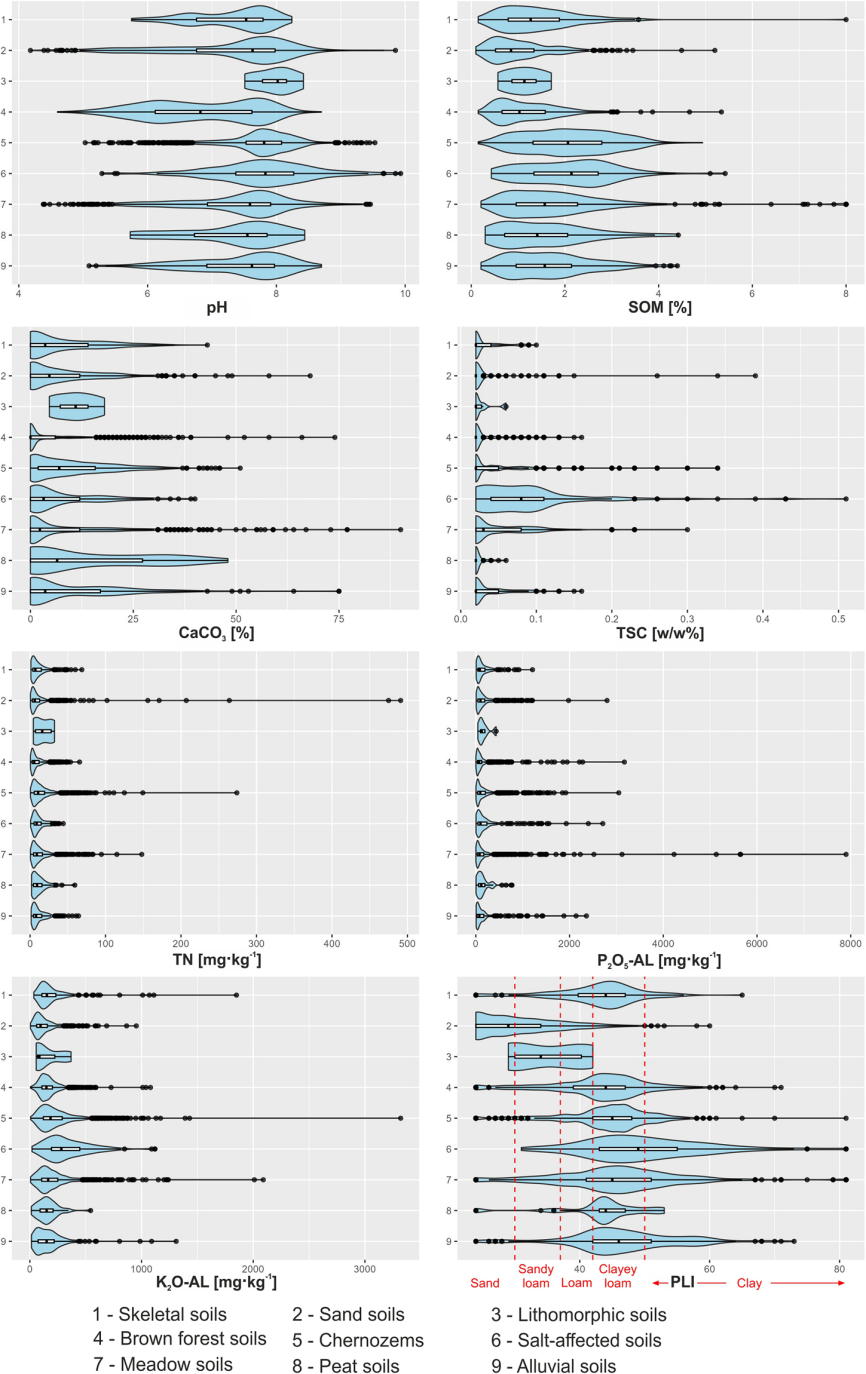
Distributions of chemical and physical soil properties of the HSDOS database. In axis Y, numbers 1–9 represent main soil types, based on the national soil-type map of Hungary^47^. Red dashed vertical lines represent the thresholds for soils with sandy, loamy and clayey textures on plasticity index (PLI) diagram according to Stefanovits *et al*.^48^.

Extended laboratory soil analysis was performed on soil samples taken from a depth of 0–30 cm, while limited laboratory soil analysis was conducted on samples taken from depths of 30–60 cm and 60–90 cm, which did not include the analysis of metallic elements and sulphate thus only the soil chemical parameters present in the limited analysis were considered during database development. Descriptive statistics and distribution of the soil laboratory data set were represented in Table 1. and Fig. 3, respectively.

**Table 1:** Comprehensive descriptive statistics of laboratory analysis data (N = 5490) for soil samples from the entire HSDOS database.

Variable	Mean	Median	Min	Max	St.Dev.	Skewness	Kurtosis
pH_KCl_	7.36	7.62	4.18	9.93	0.85	−0.82	0.27
SOM [%]	1.63	1.46	0.10	8.00	0.99	1.41	4.87
CaCO_3_ [%]	7.81	3.60	0.00	90.00	10.16	1.89	5.34
TSC [w/w %]	0.04	0.02	0.02	0.51	0.04	3.51	21.28
TN [mg kg^−1^]	12.36	8.15	0.50	491.00	16.32	11.94	284.23
P_2_O_5_-AL [mg kg^−1^]	162.72	83.75	1.50	7900.00	297.22	9.48	160.51
K_2_O-AL [mg kg^−1^]	197.08	158.00	5.10	3320.00	160.72	4.06	41.01
PLI	42.67	44.00	24.00	81.00	9.04	−0.10	0.74

### Spectral measurement

**Fig. 4:**
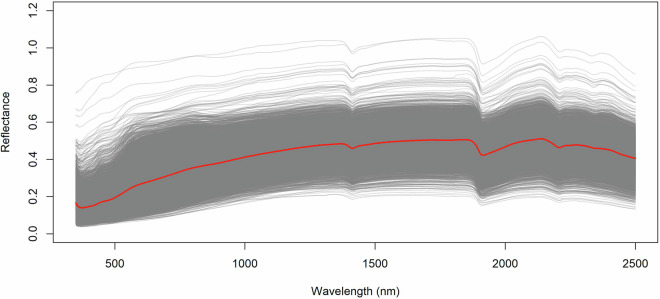
The Vis-NIR spectral reflectance after corrections and technical validation. The red line represents the mean soil spectrum.

The dried and sieved legacy soil samples of HSDOS were stored in the Institute for Soil Sciences’ soil sample bank and revisited for spectral characterization between 2022 and 2023. The subsamples of the stored soil material were measured with an Analytical Spectral Devices (currently Malvern Panalytical) FieldSpec 4 spectroradiometer in laboratory measurement configuration^28^ using an accompanying contact probe. Prior to the start of each measuring session, the spectroradiometer and the Halogen light bulb inside the contact probe was switched on and heated up for at least 1 hour to stabilize their internal temperature. In the RS3 software, the spectral recording was configured as 10/100/10 where the first value represents the number of taken white reference measurements, the second stands for the dark reference numbers and the third one is the numbers of recorded sample spectra. The software automatically calculates the mean spectrum for the white and dark references and store them temporary for the time of measurement (or until the next calibration, initiated by the user). The final spectrum for a sample is also calculated by the mean function. Because of calibration constraints, measuring sessions were divided into rounds that started with the optimization and calibration of the FieldSpec 4 using a calibrated Spectralon white reference panel, followed by the measurement of 25 soil samples and ending with a control measurement of the Spectralon panel to check for possible shifts or saturations in the reflectance data. In case of these errors present, FieldSpec 4 was recalibrated by optimization and white reference measurement prior to the next measuring round. The Vis-NIR spectra ranging from 350 nm to 2,500 nm was recorded for each soil sample based on the average of 50 measurements in five repetitions, storing reflectance data at a nominal spectral resolution of 3 nm at 700 nm and 8 nm at 1,400/2,100 nm, which is then interpolated to 1 nm uniformly to the whole range and stored in ASD file format. The raw ASD files were later converted to Excel XLSX format and merged to contain all measured data in one file. Five repetitions of the reflectance values of each soil sample were used for validation (see Technical validation section) and to calculate the representative, averaged spectrum of each HSDOS soil sample (Fig. 4).

## Data Records

The data set is available on the Zenodo online repository at 10.5281/zenodo.14610222^29^.

The full data set containing data of 5,490 samples is organized into a single CSV file, starting with column containing unique sample IDs for identification, followed by date and time of field sampling, then EOV/HD72 and geographical coordinates of the sample location. In the next section, the soil physico-chemical parameters derived by laboratory analysis were placed, closed by the unitless factor number indicating the soil profile depth of the sample, where ‘1’ refers for 0–30 cm, ‘2’ for 30–60 cm and ‘3’ for 60–90 cm profile depth. Reflectance values are stored in columns ‘SPC.n’, where ‘n’ is referring to the measured wavelength number in nanometer, between 350 and 2,500 nm with 1 nm sampling distance. Detailed description of columns with used laboratory test methods (where applicable) and units can be seen in Table 2.

**Table 2:** Summary of included attributes and data set structure with laboratory test methods applied on the soil samples.

Column name	Description	Method	Unit
SAMPLE_TDR_ID	Original TDR IDs	—	—
SAMPLING_DATE	Date of sampling	—	YYYY-MM-DD
NORTHING_EOV	Northing coordinate of sampling area centroids	—	m
EASTING_EOV	Easting coordinate of sampling area centroids	—	m
LON_WGS84	Longitude of sampling area centroids	—	°
LAT_WGS84	Latitude of sampling area centroids	—	°
pH_KCl	pH	Potentiometer (MSZ–08 0206-2: 1978)^39^	—
SOM	Soil organic matter	E4/E6 ratio^40,41^ (MSZ–08-0452:1980)^42^	%
CaCO3	Calcium carbonate	Scheibler type calcimeter (MSZ–08 0206-2:1978)^39^	%
TSC	Total salt content	EC-TDS electrode (MSZ–08-0206-2:1978)^39^	w/w %
TN	Total nitrogen	Kjeldahl method^43^ (ISO 11261:1995)^44^	mg kg^−1^
P2O5_AL	Soluble phosphorus	AL extract atomic adsorption spectrophotometry (MSZ 20135:1999)^45^	mg kg^−1^
K2O_AL	Soluble potassium	AL extract, flame photometer (MSZ 20135:1999)^45^	mg kg^−1^
PLI	Plasticity index according to Hungarian standard	Yarn test of Arany (MSZ–08 0205-2:1978)^46^	—
PROFILE_LEVEL	Soil profile depth level	—	—
SPC350:2500	spectral reflectance in the range of 350 and 2500 nm	ASD FieldSpec 4 spectroradiometer	—

## Technical Validation

Samples with invalid (i.e., out-of-range) or missing values were excluded from the laboratory data set. However, samples with extreme but valid values were retained, allowing potential users of the database to experiment with various data filtering methods and thresholds to identify potential outliers.

The raw Vis-NIR reflectance data set was validated and corrected against two different types of error related to spectral measurements. One is the splice correction to eliminate the shift in the reflectance values at 1,000 and 1,800 nm wavelengths caused by the sensor structure of the FieldSpec 4. Second is the elimination of bad repeated measurements of a soil sample with high standard deviation (SD) caused by bad contact between the contact probe and soil sample or by the saturation in the FieldSpec 4 during measurement. Therefore, the SD of the five repeated spectral measurements was calculated and evaluated against a 5% threshold (on the recorded 0–1 scale, it is equal to 0.05) on all recorded wavelengths. If the value of SD was higher than the given threshold on any wavelengths, all five spectral measurements of the soil sample was considered bad and it was excluded from the database^29^. Both spectral validation and correction processes were implemented in our workflow programmed in R^30^. A simplified version of the script, containing all main pre-processing and validation steps, is shared via Zenodo besides the main data set^29^. Details are given in the Code Availability section and in the R script file itself.

Due to the unavailability of some soil samples in the sample bank and to presented validation steps, only 5,490 samples’ data were included in the published SSL^29^.

## Usage Notes

Two further common practices exist in the use of soil spectral libraries. These are additional (mostly optional and independent) pre-treatment methods to clean the raw reflectance data and the inference of the spectral and soil properties of interest to establish statistical relationships between them using different methods. These methods vary from linear regression techniques to machine learning-based models, which have been recently developed.

The aforementioned pre-processing techniques include (1) the transformation of reflectance values to absorbance to highlight the linear relationship^31^ between chemical components and spectral wavelengths, (2) noise removal with trimming^32^ of the first and last 100 wavelengths with high saturation and noisy pattern due to laying on the boundary of the recorded range or either with smoothing of values with moving window averaging (most widely used method for this step is the Savitzky-Golay method^33^ implementing configurable differentiation and smoothing in one step). Other common methods are the scatter correction (via the use of Standard Normal Variate^34^, Multiplicative Scatter Correction^35^ functions or detrending), transformation by first or second degree derivatives^36^, and finally centering and standardization. All of the aforementioned techniques can be employed independently or in conjunction with each other in a processing pipeline as a pre-treatment method^18,36^.

## Data Availability

A simple R script was developed for the validation and pre-processing of the raw spectral measurements of the FieldSpec 4 spectroradiometer. This script implements three features: (1) the splice or shift correction to eliminate small differences in the measured reflectance values at 1,000 and 1,800 nm caused by the three independent detectors of FieldSpec 4 covering the Vis-NIR, SWIR1 and SWIR2 parts of the recorded spectrum, (2) calculation of the repetitions’ SD for all wavelength to check there is no distinctive difference (SD > 0.05) between the five spectral measurements of one soil sample and (3) averaging the repeated measurements of the corresponding soil sample. During the development process, the ‘prospectr’^37^ and ‘readxl’^38^ R packages were employed for data handling. The R script is accessible in conjunction with the data set on the Zenodo online repository at 10.5281/zenodo.14610222^29^.
